# Corticosteroids for severe CAP: the pros

**DOI:** 10.5935/0103-507X.20150041

**Published:** 2015

**Authors:** Antoni Torres

**Affiliations:** 1 Servei de Pneumologia, Hospital Clinic of Barcelona - Barcelona, Spain.; 2 Universitat de Barcelona. IDIBAPS. CIBERES - Barcelona, Spain.

Severe community-acquired pneumonia (CAP) occurs in approximately 10% of hospitalized patients with CAP, and it still carries a high morbidity and mortality. In a multicenter study on severe pneumococcal CAP, the mortality of this population was 29%, with high rates of patients requiring mechanical ventilation and a shock.^(1)^ Patients with severe CAP might die despite early and adequate antibiotic treatment, which is probably partially due to an imbalanced and disproportionate local and systemic inflammatory response that contributes to the impairment of alveolar gas-exchange, sepsis and end-organ dysfunction.^(2)^

There is no doubt that systemic adjunctive corticosteroid therapy attenuates the local and systemic inflammatory response^(3)^ and may potentially decrease *acute respiratory distress syndrome*, sepsis and mortality. In a model of *Pseudomonas aeruginosa* in mechanically ventilated piglets, we observed a lower lung bacterial burden and less severe histological pneumonia in piglets that were treated with corticosteroids plus antibiotics.^(4)^ In humans, several randomized controlled trials (RCTs) have been performed, with the participants largely being hospitalized, non-severe CAP patients. The results of these trials have been negative^(5)^ or have demonstrated a reduction in the length of stay^(6)^ or in the period required to reach clinical stability.^(7)^ Four previous studies have been performed on severe CAP.^(8-11)^ A meta-analysis that included some of these studies^(12)^ demonstrated that the pooled effect of steroids in severe CAP is a reduction in mortality.

However, in most of the RCTs, the following pitfalls are present:

1. The inclusion of patients with a low severity of CAP, i.e., PORT I to III classes, who are. These patients have a low mortality and, consequently, it would be very difficult to perform a RCT in this population with a primary end-point of mortality.
2. The inclusion of patients independent of the initial level of inflammation. According to the rationale of using steroids in CAP, a high inflammatory response is imperative. Until now, this variable has not been taken into account. In addition, patients with a high inflammatory response (such as a high C-reactive protein - CRP) have a higher rates of treatment failure^(13)^ and mortality.^(14)^
3. The dosages, type and length of treatment are very different between RCTs, which makes it very difficult to establish comparisons among them.
4. The primary end-points are different between studies. Some of them are “soft”, such as the length of stay or clinical stability. The first is very subjective and the second is driven by the abolishment of fever by corticosteroids.

We performed a RCT^(15)^ comparing methyl-prednisolone (0.5mg/kg every 12 hours for 5 days) *versus* placebo with the following important differential characteristics:

1. We only included severe CAP patients with criteria of severe CAP (major or minor modified American Thoracic Society (ATS) criteria or Pneumonia Severity Index - PSI - V).
2. We choose a threshold of 15mg/L of CRP in the blood.
3. Instead of choosing mortality as a primary end-point, we chose treatment failure. Treatment failure in CAP is associated with a higher mortality, which our group previously demonstrated. In the present study, we used a composite end-point, including early or late treatment failure.
4. We monitored the systemic inflammatory (CRP, IL6, IL8, IL10 and TNF-alpha) response until day 7 after the inclusion of a patient in the trial.

We observed a decrease from 31% to 13% in treatment failure (p = 0.02). In other words, corticosteroids reduced the risk of treatment failure with an odds ratio of 0.34. The mortality did not differ between the groups (10% in the methylprednisolone arm *versus* 15% in the placebo arm; p = 0.37). This reduction in treatment failure was more evident for late treatment failure (3 *versus* 25%; p = 0.001) and especially in radiographic progression, which is one of the variables included in the composite definition of late treatment failure (2 *versus* 15%; p = 0.007). The rates of side effects were not important and were similar between the arms.

Potential pitfalls of our study were the long-term recruitment period (8 years) and that we used methylprednisolone for only 5 days with an abrupt interruption of the treatment. However, we monitored inflammation until day 7 and did not observe a rebound of the inflammatory response.

What is the interpretation of our results?

In agreement with the editorial comment accompanying the article,^(16)^ we explain that lower rates of treatment failure, particularly late failure, and of radiographic progression can be due to stopping the progression to *acute respiratory distress syndrome *or the potential blocking of the Jarisch-Herxheimer reaction, which is thought to be due to high concentrations of cytokine release after the initiation of antibiotics. This process is possibly influenced by the release of endotoxin or other bacterial mediators in patients with a high bacterial burden, considering it also occurs in meningococcal disease.^(17)^

Having said that, it is time to start introducing corticosteroids into the clinical practice for treating severe CAP. To do so, we need to select severe CAP patients with a high inflammatory response measured by CRP. We also need to exclude patients with influenza pneumonia in our trial. It is now clear that corticosteroids increase mortality in patients with influenza pneumonia.^(18)^ We do not know what would happen in other pure viral pneumonias (adenovirus, rhinovirus, and respiratory syncytial virus). In any case, a high CRP ensures that the pneumonia is not purely viral.

The next steps are the following:

1. Investigate the potential synergies between macrolides and corticosteroids. In an animal model of pneumonia from M. *pneumoniae*, the association of macrolides and steroids was histologically beneficial.^(19)^ These investigations can be performed in animal models of pneumonia.
2. Perform a meta-analysis using individual data with a particular focus on severe CAP, as this can provide useful clinical information.

In summary, corticosteroids are useful in treating severe CAP and can help decrease treatment failure and likely mortality. The two important premises for their utilization are a high systemic inflammatory response and the elimination of influenza pneumonia.
